# Differences in Clinical Characteristics and Brain Activity between Patients with Low- and High-Frequency Tinnitus

**DOI:** 10.1155/2020/5285362

**Published:** 2020-07-26

**Authors:** Jiajia Zhang, Zhen Zhang, Shujian Huang, Huiqun Zhou, Yanmei Feng, Haibo Shi, Dan Wang, Wenya Nan, Hui Wang, Shankai Yin

**Affiliations:** ^1^Department of Otolaryngology-Head and Neck Surgery, Shanghai Jiao Tong University Affiliated Sixth People's Hospital, 600 Yishan Road, Shanghai 200233, China; ^2^Otolaryngology Institute of Shanghai Jiao Tong University, Shanghai 200233, China; ^3^Shanghai Key Laboratory of Sleep Disordered Breathing, Shanghai 200233, China; ^4^Department of Radiology, Shanghai Jiao Tong University Affiliated Sixth People's Hospital, Shanghai 200233, China; ^5^Department of Psychology, Shanghai Normal University, Shanghai 200233, China

## Abstract

This study was aimed at delineating and comparing differences in clinical characteristics and brain activity between patients with low- and high-frequency tinnitus (LFT and HFT, respectively) using high-density electroencephalography (EEG). This study enrolled 3217 patients with subjective tinnitus who were divided into LFT (frequency < 4000 Hz) and HFT (≥4000 Hz) groups. Data regarding medical history, Tinnitus Handicap Inventory, tinnitus matching, and hearing threshold were collected from all patients. Twenty tinnitus patients and 20 volunteers were subjected to 256-channel EEG, and neurophysiological differences were evaluated using standardized low-resolution brain electromagnetic tomography (sLORETA) source-localized EEG recordings. Significant differences in sex (*p* < 0.001), age (*p* = 0.022), laterality (*p* < 0.001), intensity (*p* < 0.001), tinnitus type (*p* < 0.001), persistent tinnitus (*p* = 0.04), average threshold (*p* < 0.001), and hearing loss (*p* = 0.028) were observed between LFT and HFT groups. The tinnitus pitch only appeared to be correlated with the threshold of the worst hearing loss in the HFT group. Compared with the controls, the LFT group exhibited increased gamma power (*p* < 0.05), predominantly in the posterior cingulate cortex (PCC, BA31), whereas the HFT group had significantly decreased alpha1 power (*p* < 0.05) in the angular gyrus (BA39) and auditory association cortex (BA22). Higher gamma linear connectivity between right BA39 and right BA41 was observed in the HFT group relative to controls (*t* = 3.637, *p* = 0.027). Significant changes associated with increased gamma in the LFT group and decreased alpha1 in the HFT group indicate that tinnitus pitch is crucial for matching between the tinnitus and control groups. Differences of band frequency energy in brain activity levels may contribute to the clinical characteristics and internal tinnitus “spectrum” differences.

## 1. Introduction

Tinnitus is characterized by the perception of an auditory phantom, such that patients perceive auditory sensations in the absence of any external sound source [1]. This condition is increasingly prevalent in both young (16.0–20.5%) and elderly populations (30%) [2, 3]. Tinnitus is commonly described as a ringing, buzzing, cricket-like, hissing, whistling, or humming sound or as a combination of these sounds [4]. The perceived sound may be soft or loud, a low- or high-pitched tone or noise, and intermittent or constant. Although most patients manage their tinnitus well, severe cases are always accompanied by other symptoms such as annoyance, anxiety, depression, insomnia, and cognitive dysfunction [5–7].

Currently, the severity of this condition is evaluated by a series of psychoacoustic tests and evaluation scales, including pitch matching (PM), loudness matching (LM), minimal masking levels (MMLS), gap detection (GAP), residual suppression (RI), and the Tinnitus Handicap Inventory (THI) [8–11]. However, patients with tinnitus show significant heterogeneity, which mainly presents as different characteristics of sound and varying degrees of accompanying symptoms. Approximately 80% of the individuals with tinnitus have accompanying hearing loss [12]. The correspondence between the frequency of tinnitus and the frequency range of hearing loss seems to indicate the correlation between the deprivation of auditory input and tinnitus generation [13]. Nevertheless, this correspondence was mainly validated in the patients with high-frequency tinnitus, and the definite rates of correspondence at different frequency tinnitus subgroups were not clearly investigated in a large sample [12].

Indeed, tinnitus is always accompanied by a cortical reorganization due to the hearing loss [14]. Previous studies suggested a strong positive association between the subjective strength of tinnitus and the magnitude of the shift in the tinnitus frequency in the auditory cortex [15]. Other studies indicated that tinnitus results from changes in the firing patterns of neurons in the central auditory system and from changes in burst firing and neural synchrony [16]. These results suggest a potential correlation between spontaneous neural activity and tinnitus, as well as a causal link between the characteristic frequency that dominates the reorganized neural map and the tinnitus pitch [17].

High-density electroencephalography (HD-EEG), which yields data with a high temporal resolution and reasonable spatial resolution, has been identified recently as a powerful tool for studies of dynamic brain activity [16, 18, 19]. EEG enables the noninvasive reconstruction of a region of interest (ROI) via the application of source analysis methods to scalp-recorded neuronal activities [20–22]. A previous EEG-based study identified differences in the delta, beta, and gamma-frequency brain activity bands between patients with narrowband noise tinnitus and pure-tone tinnitus [23]. This type of tinnitus pitch assessment is significant not only in terms of the systematic documentation of patients' symptoms but also for monitoring the impacts of interventions and treatment planning strategies involving acoustic stimulation, such as tinnitus maskers or transcranial magnetic stimulation [16, 24, 25].

Therefore, we aimed herein to determine the internal tinnitus “spectrum” by identifying the various pitch components that contribute to the overall tinnitus sensation. Moreover, we used source-localized resting-state EEG recordings to explore potential relationships between the detailed aspects of this spectrum (high frequency versus low frequency) and neurophysiological differences between tinnitus patients and control subjects.

## 2. Methods

### 2.1. Participants

The clinical data were collected from outpatients with subjective tinnitus who visited our tinnitus clinic at the Otolaryngology-Head and Neck Surgery Department of the Sixth People's Hospital affiliated with Shanghai Jiao Tong University between May 2016 and December 2018. Patients with subjective tinnitus who were symptomatic at the time of evaluation were included in this study. To increase the sample homogeneity, the following individuals were excluded from the study: patients with significant mental health problems, tinnitus with pulsatile tinnitus due to aberrant vascular malformation, Meniere's disease, otosclerosis, chronic headache, neurological disorders (e.g., brain tumors), and traumatic brain injury or stroke and those receiving treatment for mental disorders. Patients whose pitch of tinnitus could not be matched were also excluded. Patients with tinnitus frequency lower than 4 kHz were included in the low-frequency tinnitus (LFT) group; patients with tinnitus frequency higher than or equal to 4 kHz were included in the high-frequency tinnitus (HFT) group.

Subsequently, HD-EEG was performed on 40 participants, including 20 healthy volunteers (mean age: 38.28 ± 15.9 years; 40% men, 60% women) and 20 patients with tinnitus (mean age: 36.3 ± 11.64 years; 40% men, 60% women) who were also divided into LFT and HFT groups. Based on previous studies, EEG results of patients with tinnitus can be affected by many factors such as sex [26], the laterality of tinnitus [20], the duration of tinnitus [27], tinnitus type [23], or pure-tone threshold [13]. Consequently, those factors were matched in our study, and there were no statistically significant differences between the LFT and HFT groups regarding these parameters (Table 1). Twenty healthy volunteers were included as the control group and were matched for age, sex, and hearing threshold.

This study was approved by the Institutional Ethics Review Board of Shanghai, the Sixth People's Hospital affiliated with Shanghai Jiao Tong University, and was registered with the Chinese Clinical Trial Registry (Registration number: ChiCTR-INR-16008092). Potential consequences and benefits of the study were explained, and written informed consent was obtained from all participants before inclusion in the study.

### 2.2. Auditory Testing and Tinnitus Matching

All baseline evaluations and tests were performed by qualified medical assistants in a soundproof room. Audiograms were measured in 1-octave steps at frequencies ranging from 0.25 to 8 kHz using a manual audiometer (GSI-61, Grason-Stadler Inc., Eden Prairie, MN, USA) coupled with TDH-39 headphones. Tympanograms were obtained over a pressure range of 200 to −400 daPa at 226 Hz using a GSI tympanometer (TympStar, Grason-Stadler Inc.). The passing criteria were a type A peak in the range of −100 to +50 daPa and a static admittance of 0.3–1.6 mho.

The loudness and pitch of tinnitus were matched using a Tinnilogic™ BTD02 audiometer (Betterlife Medical Co., Ltd., Jiangsu, China) in a soundproof room. Matching was performed in a closed field. The participants were asked to concentrate on the dominant pitch of the tinnitus, and the external sound was adjusted by the tester to match the tinnitus in terms of loudness, frequency, and affected side.

### 2.3. Measurement of Tinnitus Severity

The 25-item beta version of the THI was used as a subjective measure of the handicap experienced due to tinnitus [28]. The participants were instructed to respond with yes (4 points), sometimes (2 points), or no (0 point) for each item on the inventory. These responses were added, with the total score ranging from 0 to 100 points. Depending on the total score, the handicap caused by tinnitus could be classified as slight, mild, moderate, severe, or catastrophic.

### 2.4. EEG Recording and Data Preprocessing

Spontaneous EEG signals were collected in a soundproof room. All participants were asked to sit upright on a chair in a comfortable position after abstaining from alcohol and caffeinated beverage consumption for 24 h prior to the recording. The EEG was recorded of each participant for 5 min with the eyes closed. EEG data were recorded with 256 channels on EGI's HydroCel Geodesic Sensor Net, and Cz was used as the reference channel. The electrode-skin impedance was controlled at <50 k*Ω* for each channel. The participants were asked to remain awake and keep their eyes closed. The following settings were used: sampling rate of 1000 Hz, amplification of 20 times, and band-pass filtering between 0.15 and 200 Hz.

The offline EEG analysis was conducted using custom scripts and the EEGLAB toolbox [29] on the MATLAB platform (MathWorks, Natick, MA, USA). First, the EEG signals on the scalp were band-pass filtered between 0.5 and 70 Hz while using a 50 Hz notch filter. The signals were then resampled at 500 Hz and segmented into 3 s epochs for EEG recording. Subsequently, the electrooculogram and electromyogram artifacts were corrected automatically using the blind source separation-based electrooculogram correction procedure [30] and canonical correlation analysis correction method, respectively, [31] available in the automatic artifact removal plug-in [30].

### 2.5. Scalp EEG Power Calculation

For each participant, the power spectrum density, expressed as 10∗log10 (*μ*V^2^/Hz), was computed by the spectopo function provided by EEGLAB, using Welch's method with the Hamming window, and then transformed to power spectrum density units in *μ*V^2^/Hz. Based on previous research on tinnitus [32–34], this study focused on the frequency bands including delta (2–3.5 Hz), theta (4–7.5 Hz), alpha1 (8–10 Hz), alpha2 (10–12 Hz), beta1 (13–18 Hz), beta2 (18.5–21 Hz), beta3 (21.5–30 Hz), and gamma (30.5–44 Hz). Since anatomical and neurophysiological properties of the brain, cranial bone structure, and electrode impedances [35] can influence the absolute EEG power, the relative power of each frequency band was computed by the mean power of each band divided by the mean power of 2-45 Hz. Finally, the relative power in each frequency band was averaged across all electrodes for further statistical analysis.

### 2.6. Source Localization

Standardized low-resolution brain electromagnetic tomography (sLORETA) is a genuine inverse solution that enables exact localization with zero error in the presence of a measurement and structured biological noise [36]. We used the method recommended by the developers of KEY-LORETA software (publicly available free at http://www.uzh.ch/keyinst/loreta.htm) to estimate the locations of the sources of the electrical potentials recorded on scalp EEG. Here, the artifact-free EEG epochs were exported in the ASCII format from MATLAB to LORETA software. The sLORETA analysis included the following steps: (1) computation of the sLORETA transformation matrix, (2) calculation of EEG crossspectra in the eight abovementioned frequency bands, and (3) computation of the three-dimensional (3D) cortical distribution of the electric neuronal generators for each frequency band.

### 2.7. Functional Connectivity

In general, functional connectivity can be expressed by the coherence and phase synchronization between time series corresponding to different spatial locations. However, any measure of dependence is highly contaminated with an instantaneous, nonphysiological contribution because of the volume conduction and low spatial resolution [37]. To solve this problem, Pascual-Marqui proposed a new technique that considerably abrogated this confounding factor [38]. Furthermore, this measure of dependence can be applied jointly to any number of brain areas (i.e., distributed cortical networks) for which the activity can be estimated using sLORETA. Consequently, nonnegative measures of linear dependence (i.e., coherence) between the multivariate time series are defined. These measures yield a zero value only in the presence of independence of the pertinent type.

Based on this principle, the lagged linear connectivity was calculated. Five bilateral ROIs were defined based on the present findings and source analysis and previous brain research related to tinnitus: (1) the secondary auditory cortex (BA21, BA22), (2) posterior cingulate cortex (BA23, BA31), (3) angular gyrus (BA39), (4) intraparietal sulcus (BA40), and (5) primary auditory cortex (BA41, BA42) [22, 32].

### 2.8. Statistics

**Figure 1:**
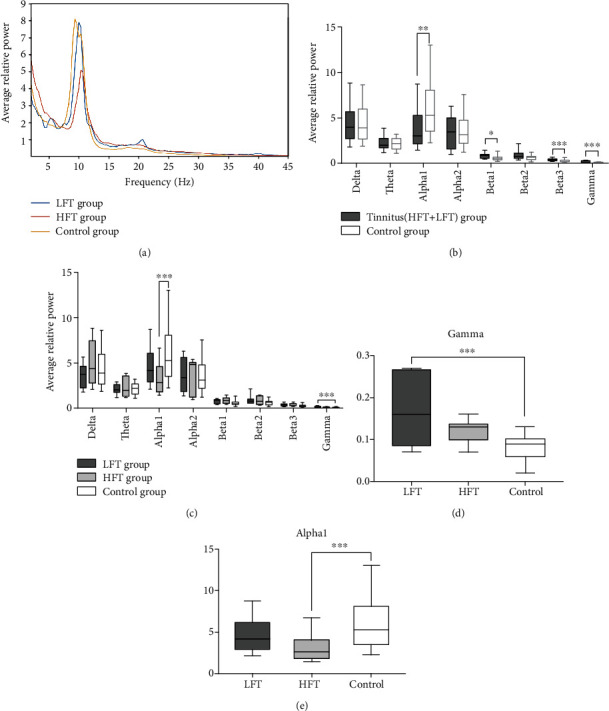
EEG results of the tinnitus and control groups: (a) the distribution of averaged brain activities along the band frequency in each group; (b) comparisons of average EEG power at eight frequency bands between the total tinnitus group and control groups; (c) intergroup comparisons of averaged brain activities; (d, e) the magnified view at gamma (30.5–44 Hz) and alpha1 (8–10 Hz) frequency bands in (c). ^∗^*p* < 0.05, ^∗∗^*p* < 0.01, and ^∗∗∗^*p* < 0.001 against the control group.

The chi-squared test and *t*-test were used to determine intergroup differences (Tables 1 and 2) depending on the data type. These calculations were performed using SPSS version 24 (SPSS/PC, Chicago, IL, USA). The power spectra of groups were compared using two-way repeated-measurement ANOVAs, followed by post hoc tests (Holm-Sidak) for each frequency point (Figure 1) in SPSS. sLORETA was used to perform between-condition voxel-by-voxel comparisons of the current density distributions, which were then used to identify potential differences in brain electrical activity among the three groups. Nonparametric statistical analyses of functional sLORETA images (i.e., statistical nonparametric mapping (SnPM)) were performed for each contrast; here, a *t*-statistic corrected for multiple comparisons was used for unpaired groups (*p* < 0.05). As explained by Nichols and Holmes, the SnPM methodology does not require any assumption of Gaussianity and corrects for all multiple comparisons [39]. We performed a voxel-by-voxel test (comprising 6239 voxels each) for each different frequency band. For all analyses, a *p* value < 0.05 was considered to indicate statistical significance.

## 3. Results

### 3.1. Patient and Demographic Characteristics

**Figure 2:**
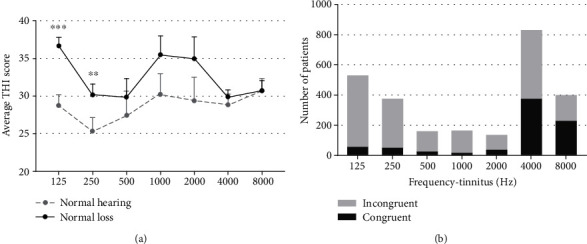
(a) Comparison of the THI scores of tinnitus patients with normal and abnormal hearing. ^∗∗^*p* < 0.01 and ^∗∗∗^*p* < 0.001 for differences between the labeled group at each frequency versus the normal hearing group. Error bars represent the standard errors of the means. (b) The number of patients with or without coincidence (tinnitus pitch vs. hearing threshold showed the deepness of hearing loss) in various frequency tinnitus subgroups with hearing loss.

The tinnitus pitches reported by patients ranged from 0.25 to 12 kHz, and 55.4% and 44.6% of subjects were classified into the LFT and HFT groups, respectively. The characteristics of the LFT and HFT groups are displayed in Table 2. Notably, these groups differed significantly with respect to sex (*p* < 0.001), with a significantly higher proportion of women in the LFT group. A significantly higher tinnitus intensity was also observed in the LFT group (*p* < 0.001). Significant intergroup differences were also observed with respect to age (*p* = 0.046), laterality (*p* < 0.001), tinnitus type (*p* < 0.001), tinnitus persistence (*p* = 0.04), average threshold (*p* < 0.001), and hearing loss (*p* = 0.028). However, THI scores did not differ significantly for the two groups (*p* = 0.062). Patients with hearing loss had a higher THI score than those with normal hearing, and this was especially notable among LFT patients who reported tinnitus frequencies of 125 Hz (*p* < 0.001) and 250 Hz (*p* = 0.03). However, we did not observe a significant difference in the THI scores between patients with and without hearing loss in the HFT group (Figure 2(a)). The tinnitus pitch was most commonly matched to high frequencies, at which the hearing threshold indicated the depth of hearing loss among the 3217 patients. However, no correlation was observed between tinnitus pitch and worst threshold in the LFT group (Figure 2(b)).

### 3.2. EEG Results

**Figure 3:**
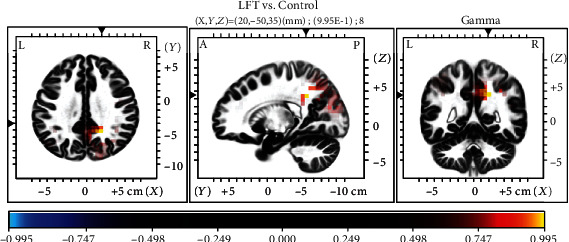
Comparison of sLORETA results between the low-frequency tinnitus (LTF) and control groups. Note the relative increase in gamma activity in the posterior cingulate cortex in patients with LTF.

Figure 1 shows the EEG results of the tinnitus and control groups. The power spectrum densities recorded across all scalp electrodes in each group were averaged to show the distribution of brain activities along the frequency ranging from delta (2–3.5 Hz) to gamma (30.5–44 Hz) (Figure 1(a)). Significant power differences were observed at alpha1 (*t* = 3.15, *p* < 0.01), beta1 (*t* = 2.55, *p* < 0.05), beta3 (*t* = 2.62, *p* < 0.01), and gamma (*t* = 3.92, *p* < 0.001) bands when we compared the total tinnitus group (including the LFT and HFT groups) with the control group (Figure 1(b)). However, when we compared the subgroup with the control group, only the alpha1 and gamma band showed significant changes in the LFT and HFT groups, respectively. (Figure 1(c)). More specifically, pairwise comparisons showed that (1) the LFT group demonstrated a significantly higher level of gamma power (*t* = 3.63, *p* < 0.001) (Figure 1(d)), (2) and the HFT group had a significant decrease in alpha1 frequency band (*t* = 3.71, *p* < 0.001) (Figure 1(e)), (3) but no significant difference was found between the LFT and HFT groups across the entire frequency bands (*t* = 0.58, *p* = 0.563).

### 3.3. Source Localization Results

**Figure 4:**
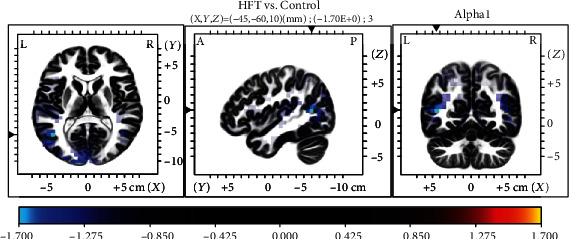
Comparison of sLORETA results between the HFT and control groups. Note the relative decreases in alpha1 activity in the angular gyrus and second auditory cortex in patients with HFT.

The sLORETA analysis revealed no significant differences between the LFT and HFT groups. In the LFT group, we observed greater gamma activity in the posterior cingulate cortex (PCC, BA31) relative to the levels in the control group (Figure 3). A synchronized decreased alpha1 activity was observed predominantly in the angular gyrus (BA39) and secondary auditory cortex (BA 22) in the HFT group in comparison to that in the control group (Figure 4).

### 3.4. Functional Connectivity

**Figure 5:**
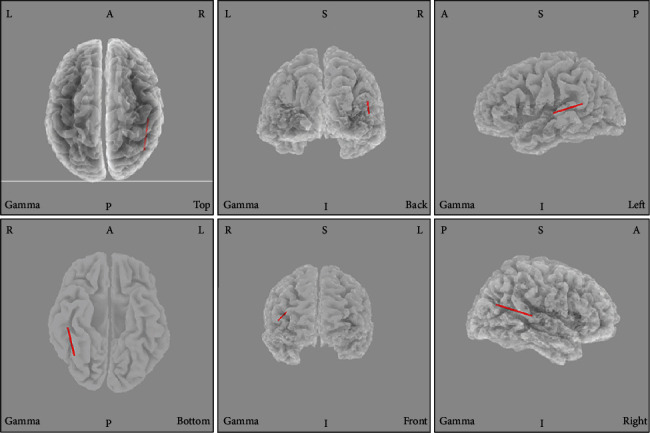
Comparison of functional connectivity between the high-frequency tinnitus (HFT) and control groups in sLORETA source space. Higher gamma linear connectivity between the right angular gyrus (BA39) and right primary auditory cortex (BA41) was observed in the HFT group relative to the control group.

Increased gamma linear connectivity between the right BA39 and right BA41 was observed in the HFT group relative to that in the control group (*p* = 0.027). No statistical differences between the default mode functions and networks were observed between the LFT group and the control group. Similarly, there were no statistically significant differences in these parameters between the HFT and LFT groups (Figure 5).

## 4. Discussion

In the mammal's inner ear, hair cells and spiral ganglion neurons are critical for hearing ability; hair cells convert the mechanical sound waves into neural signals, and spiral ganglion neuron transmits these signals to the auditory cortex for hearing [40–42]. In the mammal's inner ear, hair cells and spiral ganglion neurons are vulnerable for multiple damages, including gene mutation, noise, different ototoxic drugs, inflammation, or aging [43–47] while the mammals only have very limited hair cell and spiral ganglion neuron regeneration ability; most of the damaged hair cells and spiral ganglion neurons cannot be spontaneously regenerate [48–55]. Thus, most of the hearing loss is irreversible; and usually, tinnitus is always accompanied with hearing loss. Tinnitus is characterized by an auditory phantom perception in the absence of any physical sound source, and by far pathophysiological mechanisms is still not clear. In this study, we explored and compared the characteristics of the neural activities associated with LFT and HFT. We used an sLORETA-based source analysis of resting-state EEG data to further investigate the pathophysiology of phantom sound perception in patients with tinnitus. Notably, the comparison of patients with LFT and HFT demonstrated several significant differences with respect to sex, age, laterality, intensity, tinnitus type, persistent tinnitus, hearing loss, and comorbid diseases. Moreover, patients with hearing loss had higher THI scores than those with normal hearing in the LFT group, whereas no such difference was observed in the HFT group. Moreover, the tinnitus pitch was correlated with high frequencies associated with the greatest hearing losses in patients with HFT, but not in those with LFT. Our EEG results revealed no significant differences in EEG power between the tinnitus groups. However, significant differences were observed between the control group and each tinnitus group. Compared with the control group, the HFT group demonstrated a significant decrease in alpha1 power, and the LFT group exhibited a significant increase in gamma power. We further used sLORETA to identify the dominant brain areas associated with these differences in EEG power. Our findings suggest that differences in brain activity levels may contribute to the observed intergroup differences in characteristics.

Tinnitus is a highly heterogeneous condition with respect to the characteristics of the perceived sound, and it is associated with various degrees of associated awareness and distress, duration, and comorbidities [56, 57]. This variability would be expected in clinical presentation to be reflected by a similar variability in the structures and functions of neuronal correlates. Therefore, it is extremely challenging to identify the underlying neuronal mechanisms of tinnitus, particularly given the high level of inconsistency among previous studies. For example, the variables that must be matched between the tinnitus and control groups remain unclear [14, 58, 59]. In this study, we matched patients in the LFT and HFT groups with respect to several demographic and clinical characteristics before exploring the underlying neurophysiological mechanisms, based on the findings of our study (Table 2). The assessment of tinnitus pitch is significant not only for the systematic documentation of patients' symptoms but also for monitoring the impacts of interventions and for treatment planning involving acoustic stimulation. In previous studies, the suppressive effect of repetitive transcranial magnetic stimulation was moderated by tinnitus type and laterality, tinnitus-related distress, and tinnitus duration, such that patients presenting with unilateral pure-tone tinnitus had significantly worse outcomes than those with noise-like tinnitus [60, 61]. Currently, no sound theory has been proposed to explain the differential effect of burst transcranial magnetic stimulation on pure-tone and noise-like tinnitus. Moreover, studies are increasingly providing strong evidence supporting the efficacy of sound therapy, during which appropriate external sounds matching the tinnitus frequency can diminish or even render tinnitus inaudible [62]. These findings suggest potential differences in the neurophysiological mechanisms underlying different tinnitus pitch types. The mechanism that may cause increasing annoyance in a patient with LFT and hearing loss remains unclear (Figure 2). However, different auditory modalities are thought to be coded by different mechanisms and spatially separate brain networks [63]. Thus, it can be assumed that different perceptual characteristics of tinnitus (e.g., pitch and loudness) might also be coded by spatially and functionally parallel and overlapping brain networks.

The alpha rhythm may indicate cortical inhibition in an EEG, as it inhibits cell assemblies from entraining to visual stimuli and is correlated with reduced metabolic activity [64–66]. Most [67–70] resting-state MEG and EEG measurements from the temporal cortex of individuals with tinnitus reveal a reduction in alpha power (8–12 Hz) and increase in slow-wave power (delta and theta, 1–6 Hz) and gamma power (>30 Hz), which is consistent with our results [70, 71]. Notably, the HFT group demonstrated a significant decreased in alpha1 band power, and the LFT group demonstrated a significantly higher level of gamma power when compared with that of the control group, respectively. Therefore, a framework was proposed, which postulates that the reduction in ongoing inhibitory alpha activity in patients with tinnitus favors the synchronization of neurons in the gamma frequency range in the resting state. Consistent with this framework, tinnitus pitch is an important variable that must be matched between the tinnitus and control groups [72]. Moreover, these alterations in oscillatory power are proposed to be generated by thalamocortical dysrhythmia [69, 73]. Thalamocortical dysrhythmia is the consequence of hyperpolarization of the thalamus, which has lost input due to deafferentation, resulting in a decreased external input. In response, brain plasticity attempts to obtain missing information from the auditory cortex neighborhood due to the decrease in surrounding inhibition. Mechanistically, this attempt is mediated by deinactivation of T-type Ca^2+^ channels and the generation of low-threshold bursting, which normally occurs only during sleep [73]. On an EEG, this change is represented by a slowing of theta activity to alpha activity, which is always accompanied by crossfrequency coupling with increased beta/gamma activity [22, 74]. Although cross-sectional and longitudinal studies have consistently demonstrated abnormal spectrum activities [62, 75], the results were less focused on tinnitus pitch and oscillatory power. Notably, we observed a significant correlation between tinnitus pitch and the gamma and alpha-band activity levels in our study.

Resting-state network measurements revealed an association of tinnitus with alterations in a wide range of brain areas [76–79]. In our study, HFT was associated with increased alpha1 activity in the second auditory cortex (BA22) and angular gyrus (BA39) regions relative to that in the control group (Figure 3). BA22 is involved in auditory processing and language reception. The angular gyrus has been associated with recollection-related activity, semantic processing [80], and auditory stimulus integration. LFT was associated with decreased gamma activity in the PCC relative to that in the control group (Figure 4). Previous research has demonstrated a role for the PCC in cognitive evaluation and sensory input memorization [81]. Moreover, increased connectivity within the gamma band in the right BA39 and right BA41 was observed in the HFT group relative to the control group. In a previous study of unilateral tinnitus patients, increased synchronized activity was observed in the angular gyrus [21]. In our study, all 20 tinnitus patients who underwent EEG also had unilateral tinnitus. The angular gyrus forms strong reciprocal connections with the parahippocampal area [82] and acts as a key node in the dorsal auditory pathway, the main function of which is the transformation of auditory representations into premotor responses [83]. In addition, coactivation of the angular area with the superior premotor cortex is important in spatial localization of auditory input [84]. Gamma-band activity in the auditory cortex is necessary for conscious auditory perception [74, 85] and thus may also contribute to the perception of a phantom sound. As summarized by a previous EEG study, activation of the auditory cortex may reflect the loudness of tinnitus, while conscious perception of tinnitus, its salience, and the associated distress are associated with the coactivation of different resting-state networks, such as the frontoparietal control system, PCC, auditory associated cortex, and salience network [20, 32, 59, 75, 86]. Tinnitus shares many common features with phantom pain, particularly the involvement of a vast network of brain regions, instead of the sensory cortex alone. While such networks are incompletely understood, the general idea that phantom perceptual experiences are network phenomena has gained consensus [87]. The perception of sound itself might generate tinnitus via increased activity in the auditory pathways mediated by the interactions of auditory brain areas with nonauditory brain networks, instead of tonotopic reorganization [59].

Our study had some limitations. Tinnitus is a heterogeneous disease, and it is difficult to eliminate the various factors contributing to this heterogeneity. We note that stricter inclusion and exclusion criteria and a critical analysis of the clinical data could be applied. The results obtained from the EEG data require careful explanation because the sample sizes of our subgroups may not have been sufficiently large. Moreover, selection bias should be considered. Future studies with larger sample sizes and additional subgroup comparisons (e.g., tinnitus with or without hearing loss) are needed to investigate the characteristics and compare the differences between LFT and HFT. Such studies should focus on the definite changes in neural activities after treatment for tinnitus involving different frequencies.

## 5. Conclusions

In conclusion, we observed several significant differences in the clinical characteristics of patients in the LFT and HFT groups. Patients with LFT appeared to be more disadvantaged by hearing loss than those with HFT, as indicated by the THI scores. Moreover, the tinnitus pitch only appeared to be correlated with the threshold of the worst hearing loss in the HFT group. Our findings suggest significant differences in the power levels of the gamma and alpha1 bands between patients with tinnitus and controls, but not between patients with different tinnitus frequency levels. Differences in brain activity levels may contribute to the observed intergroup differences in characteristics.

## Figures and Tables

**Table 1:** Electroencephalogram characteristics of patients with low- and high-frequency tinnitus.

Tinnitus patients		Age (yrs)	Sex	Tinnitus laterality	THI	Tinnitus pitch	Loudness	Duration (months)	PTA (≤2 kHz)	PTA (>2 kHz)
LFT	1	36	M	L	4	350	40	7	5	6.25
	2	52	F	R	40	200	30	120	13.75	13.75
	3	29	M	R	78	150	40	3	6.25	5
	4	48	F	L	30	150	42	3	10	10
	5	41	F	R	0	500	60	96	13.75	13.75
	6	38	M	L	10	125	25	12	7.5	8.75
	7	31	F	L	12	200	45	36	11.25	11.25
	8	30	F	R	26	120	48	36	6.25	7.5
	9	24	M	L	38	120	45	3	16.25	17.5
	10	33	F	R	36	100	36	3	8.75	8.75

Mean ± SD		36.2 ± 8.8	4 M/6F	5 L/5R	27.4 ± 22.9	—	41.1 ± 9.7	31.9 ± 42.5	9.8 ± 3.7	10.25 ± 3.85
HFT	11	60	F	R	20	8000	35	6	21.25	22.5
	12	27	M	R	22	8000	5	12	6.25	7.5
	13	31	M	L	8	8000	28	6	3.75	3.75
	14	24	F	R	20	4000	55	6	0	-1.25
	15	22	F	L	6	8000	48	96	3.75	3.75
	16	55	F	R	55	8000	41	36	5	7.5
	17	26	M	L	22	8000	25	6	-2.5	-2.5
	18	53	F	L	18	4000	30	12	15	16.25
	19	40	F	R	38	8200	31	6	6.25	8.75
	20	26	M	L	34	6000	33	12	16.25	17.5
Mean ± SD		36.4 ± 14.5	4 M/6F	5 L/5R	24.3 ± 14.6	—	33.1 ± 13.6	19.8 ± 28.3	7.5 ± 7.5	8.4 ± 8.1
*p* value		0.932	0.714	0.653	0.902	—	0.134	0.306	0.387	0.492

L: left; R: right; F: female; M: male; PTA: pure-tone threshold audiometry; HFT: high-frequency tinnitus; LFT: low-frequency tinnitus; SD: standard deviation.

**Table 2:** Characteristics of patients with low- and high-frequency tinnitus.

Characteristics	Total (*N* = 3217)	Frequency of tinnitus		*p* value
		Low frequency (*N* = 1783)	High frequency (*N* = 1434)	
Sex (%) (*n*)				
Male	43.1 (1386)	35.5 (633)	52.5 (753)	<0.001
Female	56.9 (1831)	64.5 (1150)	47.5 (681)	
Age (year)				
Mean ± SD	50.45 ± 16.9	50.99 ± 16.9	49.79 ± 16.7	0.046
Laterality (%) (*n*)				
Left	32.3 (1039)	35.6 (634)	28.2 (405)	<0.001
Right	27.6 (888)	31.2 (556)	23.2 (332)	
Bilateral	38.4 (1236)	32.1 (573)	46.2 (663)	
In head	1.7 (54)	1.1 (20)	2.4 (34)	
THI				
Mean ± SD	30.88 ± 23.6	31.57 ± 24.1	30.02 ± 22.8	0.062
Intensity (dB)				
Mean ± SD	15.56 ± 6.4	16.31 ± 6.2	14.63 ± 6.6	<0.001
Duration (day)				
Mean ± SD	836.74 ± 1442.5	797.29 ± 1409.4	885.78 ± 1481.7	0.084
Tinnitus type (%) (*n*)				
Pure tone	90.4 (2908)	87.4 (1559)	94.1 (1349)	<0.001
Otherwise	9.6 (309)	12.6 (224)	5.9 (85)	
Persistent tinnitus (%) (*n*)				
Yes	82.2 (2645)	61.5 (1096)	66.4 (952)	0.004
No	17.8 (572)	38.5 (687)	33.6 (482)	
Average threshold				
Mean ± SD	30.75 ± 23.8	32.449 ± 25.2	28.64 ± 21.8	<0.001
Hearing (%) (*n*)				
Normal	34.4 (1107)	36.1 (643)	32.4 (464)	0.028
SNHL	65.6 (2110)	63.9 (1140)	67.6 (970)	
Accompanying symptoms (%) (*n*)				
Yes	42.6 (1372)	42 (748)	43.5 (624)	0.373
No	57.4 (1845)	58 (1035)	56.5 (810)	

## Data Availability

The form data used to support the findings of this study are available on request to the corresponding author: Dr. Hui Wang, Email: wangh2014@163.com.
